# Supplementary material for: A Nectin1 Mutant Mouse Model Is Resistant to Pseudorabies Virus Infection
Source: Viruses. 2022 Apr 22;14(5):874. doi: 10.3390/v14050874 (PMC9144750; doi:10.3390/v14050874)
Supplement: Supplementary file 1 [file viruses-14-00874-s001.zip › viruses-1667966-supplementary.pdf]

Supplementary figures

```
Pig      1 MARMGLAGAAGRWWGLALGLTAFFLPGAHTQVVQVNDSMYGFIGTDVVLHCSFANPLPGV 60
Cattle   1 MARMGLAGAAGRWWGLALGLTAFFLPGAQAQMVQVNDSMYGFIGTDVVLHCSFANPLPGV 60
Human    1 MARMGLAGAAGRWWGLALGLTAFFLPGVHSQVVQVNDSMYGFIGTDVVLHCSFANPLPSV 60
Mouse    1 MARMGLAGAAGRWWGLALGLTAFFLPGTHTQVVQVNDSMYGFIGTDVVLHCSFANPLPSV 60
           ***************************.::*:**************************.*
Pig     61 KITQVTWQKATNGSKQNVAIYNPAMGVSVLAPYRERVEFLRPSFTDGTIRLSRLELEDEG 120
Cattle  61 KITQVTWQKATNGSKQNVAIYNPAMGVSVLAPYRERVEFLRPSFTDGTIRLSRLELEDEG 120
Human   61 KITQVTWQKSTNGSKQNVAIYNPSMGVSVLAPYRERVEFLRPSFTDGTIRLSRLELEDEG 120
Mouse   61 KITQVTWQKASNGSKQNMAIYNPTMGVSVLPPYEKRVEFLRPSFIDGTIRLSGLELEDEG 120
           *********::******:*****:******.**.:********* ******* *******
Pig    121 VYICEFATFPAGNRESQLNLTVMAKPTNWIEGTQAVLRAKKGKDDKVLVATCTSANGKPP 180
Cattle 121 VYICEFATFPAGNRESQLNLTVMAKPTNWIEGTHAVLRARKGQDEKVLVATCTSANGKPP 180
Human  121 VYICEFATFPTGNRESQLNLTVMAKPTNWIEGTQAVLRAKKGQDDKVLVATCTSANGKPP 180
Mouse  121 MYICEFATFPTGNRESQLNLTVMAKPTNWIEGTRAVLRARKGQDDKVLVATCTSANGKPP 180
           :*********:**********************:*****:**:*:***************
Pig    181 SVVSWETHLKGEAEYQEIRNPNGTVTVISRYRLVPSREDHRQSLACIVNYHMDRFRESLT 240
Cattle 181 SVVSWETRLKGEAEYQEIRNPNGTVTVISRYRLVPSREAHRQSLACIVNYHMDRFWESLT 240
Human  181 SVVSWETRLKGEAEYQEIRNPNGTVTVISRYRLVPSREAHQQSLACIVNYHMDRFKESLT 240
Mouse  181 SAVSWETRLKGEAEYQEIRNPNGTVTVISRYRLVPSREAHRQSLACIVNYHLDRFRESLT 240
           *.*****:****************************** *:**********:*** ****
```

Figure S1. Conservation of Nectin1 F129 residues across different mammalian species. Sequences were aligned with CLUSTALW. F129 residues are shown in red letters. Identical residues in all aligned sequences are denoted by asterisks.

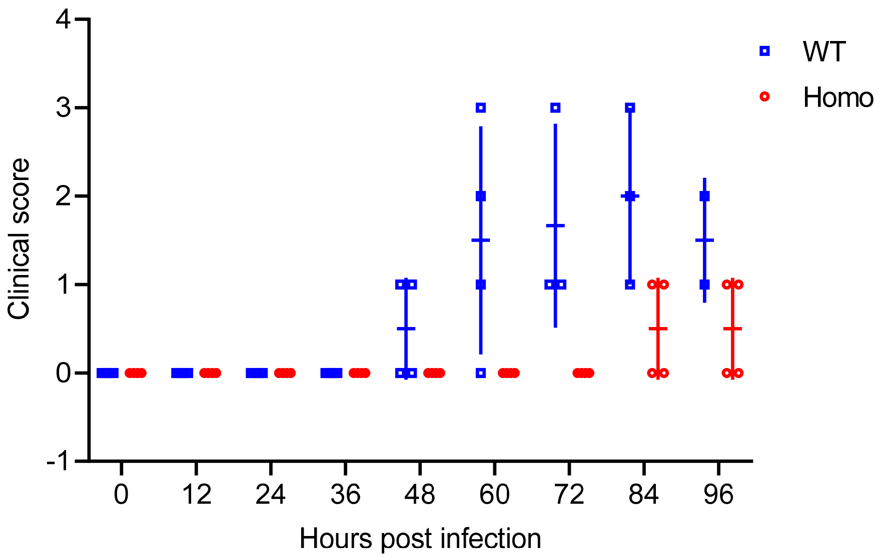

Figure S2. Clinical score for WT and homozygous (Homo) nectin1 F129A mutant mice following PRV infection in experiment 1.

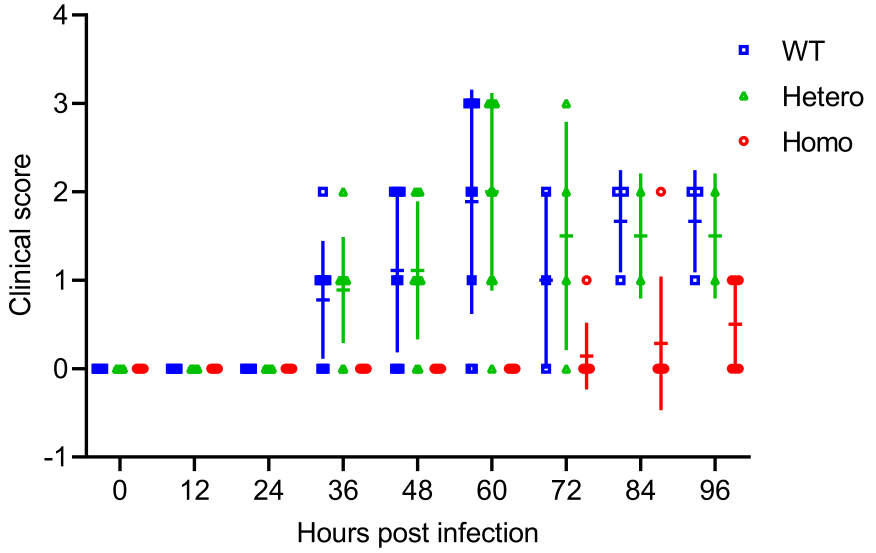

Figure S3. Clinical score for WT, heterozygous (Hetero), and homozygous (Homo) nectin1 F129A mutant mice following PRV infection in experiment 2.
